# Treatment Adherence in Patients with Obstructive Pulmonary Diseases

**DOI:** 10.3390/ijerph191811573

**Published:** 2022-09-14

**Authors:** Henryka Homętowska, Natalia Świątoniowska-Lonc, Jakub Klekowski, Mariusz Chabowski, Beata Jankowska-Polańska

**Affiliations:** 1 Specialistic Hospital of the Ministry of Interior and Administration, 40 Karłowicza Street, 48-340 Głuchołazy, Poland; 2 Innovation and Research Center, 4th Military Teaching Hospital, 5 Weigla Street, 50-981 Wrocław, Poland; 3 Student Research Group No. 180, Faculty of Medicine, Wroclaw Medical University, 50-367 Wrocław, Poland; 4 Department of Surgery, 4th Military Teaching Hospital, 5 Weigla Street, 50-981 Wrocław, Poland; 5 Division of Anaesthesiologic and Surgical Nursing, Department of Nursing and Obstetrics, Faculty of Health Science, Wroclaw Medical University, 5 Bartla Street, 51-618 Wrocław, Poland

**Keywords:** treatment adherence, compliance, chronic obstructive pulmonary disease, beliefs

## Abstract

COPD is the third most common cause of death globally. Adherence rates in patients with obstructive pulmonary diseases usually range between 10% and 40%. The aim of the study was to evaluate the level of treatment adherence to inhaled therapy in patients with obstructive pulmonary diseases. A total of 325 patients, of mean age 63.04 ± 11.29, with COPD or asthma, were included into the study between 2020 and 2021. The following questionnaires were used: Beliefs about Medicines Questionnaire, Test of Adherence to Inhalers and Adherence to Refills and Medications Scale. The respondents tended to be convinced of the necessity of their medication (3.87 points per question). The patients reported moderate levels of overall adherence (21.15 ± 6.23). A total of 74% of patients demonstrated sporadic non-compliance. We conclude that patients with obstructive pulmonary diseases are moderately adherent to their medication. Beliefs about medicines have a significant impact on adherence to medications. Being unemployed, being a non-smoker and belief in the necessity of medication are independent determinants of better medication adherence. The number of hospital admissions due to exacerbations of the disease over the last year and belief that medicines are harmful are independent determinants of poorer medication adherence.

## 1. Introduction

Chronic non-infectious respiratory diseases are one of the major causes of death both worldwide and in EU countries. The conditions leading to acute chronic respiratory failure or its exacerbation include COPD and asthma, which are among the most common chronic respiratory diseases. According to the World Health Organization, COPD affects up to one in ten adults over the age of 40 [1]. It is estimated that there are 2 million people living with COPD in Poland [2]. Asthma is estimated to affect around 235 million people worldwide. According to the 2014 ECAP (Epidemiology of Allergic Diseases in Poland) study, the prevalence of asthma in adults in Poland is 9% [2]. According to WHO statistics, COPD is currently the third most common cause of death globally (after cardiovascular disease and cancer) [1]. The effective treatment of obstructive pulmonary diseases should be multi-faceted and involve both pharmacological and non-pharmacological modalities, including the treatment of symptoms, reduction in risk factors, health education, training in self-assessment skills and provision of basic information about the condition.

Medication adherence is a pre-requisite for the successful treatment of patients with obstructive pulmonary diseases. Non-adherence to treatment leads to an exacerbation of disease symptoms and increased frequency of medical consultations and hospital admissions. Moreover, it is associated with sickness absence and higher costs associated with treating the consequences of failure to adhere to medication [3]. Data from observational studies indicate that adherence rates in patients with obstructive pulmonary diseases usually range between 10% and 40% [4] and that non-compliance is the most common cause of exacerbations, which indicate whether or not treatment is effective.

Despite the availability of modern treatment options, patients do not achieve the benefits of treatment due to the barriers to adherence they experience. A number of factors interfering with adherence depend on the individual characteristics of a given patient. However, they also depend on the patient’s environment, type of treatment and level of cooperation between the patient and their physician. The most common and important forms of non-adherence are omission of a dose of medication and early discontinuation of treatment. Among the factors having an impact on treatment adherence are beliefs about medicines, including beliefs about the necessity of medication, beliefs about the overuse of medicines and their harmfulness, as well as concerns about medicines [5]. Patients do not usually believe in the necessity of medications that are used to extend life or for secondary disease prevention and are not just for symptom relief [6]. Some patients have concerns about the long-term use and potential adverse side-effects of medicines, particularly in medicines prescribed to prevent future complications [6]. Patients who are older, have chronic conditions and use multiple medications, in particular, demonstrate different levels of willingness and capability to adhere to medications. Their attitude to treatment, as well as their beliefs and concerns are highly likely to have a significant impact on their adherence to their treatment plan. Older patients, in particular, may hold strong beliefs and views about their medication, which are often based on their own experience or the experience of their family or friends. Studies have shown that patients aged over 60 are more concerned about the risk of relatively rare serious side effects than about the risk of common mild side effects [7].

Although an increasing number of factors that have an impact on adherence are being identified, further research is necessary in order to identify new risk factors [8,9]. Even in the case of deliberate non-adherence, the focus is on such factors as the negative aspects of treatment and its adverse side-effects, which may raise patients’ concerns about using their prescribed medication. It seems that understanding the mechanisms that shape such beliefs and their relationship with adherence is extremely important for clinical practice. There is limited evidence in the literature on the relationship between beliefs about treatment and adherence in COPD and asthma patients. Krauskopf et al. found a significant association between patients’ specific concerns about medicines and medication adherence [10]. In a study by Brandstetter et al., specific beliefs about the necessity of medication were positively associated with medication adherence both in patients with asthma and in patients with COPD, whereas general beliefs about the harmfulness and over-prescription of medicines were negatively associated with medication adherence in asthma patients only [11]. In a study by Duarte-de-Araújo, the mean BMQ—Necessity score was higher in those COPD patients who adhered to their inhalation therapy [12].

Given that few publications focus on the assessment of beliefs about medicines or the relationship between those beliefs and non-adherence, the aim of the present study was to evaluate the level of treatment adherence to inhaled therapy in patients with obstructive pulmonary diseases, as measured by the Adherence to Refills and Medications Scale (ARMS) and the Test of Adherence to Inhalers (TAI), with relation to the Beliefs about Medicines Questionnaire (BMQ).

## 2. Materials and Methods

In the study period (November 2020–December 2021), 378 patients with COPD or asthma were being treated at the pulmonology outpatient clinic. Thirty-five of those patients did not meet the inclusion criteria and 12 patients refused to take part in the study. Therefore, 331 patients were included in the study and received questionnaires. During the study, 6 patients withdrew without giving a reason or failed to correctly complete questionnaires. The study group ultimately included 325 patients with COPD or asthma (51.08% of whom were men). The mean age of the patients was 63 ± 11 years. Study qualification was conducted by a trained team comprising two specialist nurses. All the qualified patients completed standardised questionnaires following their appointment with the clinic. Sociodemographic data were obtained from the medical register and are presented in Table 1. All the patients were informed about the study process and methods and about their right to withdraw from the study at any time. All the patients provided informed written consent to participate in the anonymous study.

The inclusion criteria were as follows: provision of informed written consent to take part in the study, diagnosis of an obstructive pulmonary disease (asthma or COPD), patients aged over 18, patients who had been using at least one inhaled medication for at least 6 months, cognitive status sufficient to understand the purpose and methods of the study and complete questionnaires (Mini Mental State Examination ≥18).

The following standardised questionnaires were used in the study:

- Beliefs about Medicines Questionnaire (BMQ), which is used to assess patients’ beliefs about medicines. The questionnaire consists of four domains assessing respondents’ beliefs about the overuse of medicines by doctors, beliefs about the harmfulness of medicines, beliefs about the necessity of medication and concerns about medicines. Responses are given on a 5-point Likert scale ranging from 1 = strongly disagree to 5 = strongly agree. Item scores obtained in each subscale are summed to give a total subscale score. Higher scores indicate stronger beliefs about medicines [9]. Both the original and the Polish versions of the questionnaire demonstrated satisfactory psychometric properties (Cronbach’s alpha: 0.6–0.78 and 0.64–0.82, respectively) [13].
- Test of Adherence to Inhalers (TAI), which is used to evaluate adherence to inhaled medications in patients with asthma and COPD. The questionnaire consists of 12 questions assessing sporadic, deliberate and unconscious non-compliance. The 12-item TAI includes additional items nos. 11 and 12, which are scored as 1 or 2 (where 1 = bad and 2 = good), with a score range from 2 to 4, which were designed to identify two possible causes of unconscious non-compliance. Sporadic and deliberate patterns of non-compliance were defined in the presence of scores ≤ 24 for items #1 to #5 and items #6 to #10, respectively. Unconscious non-compliance was defined in the presence of a score of 1 in at least one of the last two items (#11 and #12) of the questionnaire. Cronbach’s alpha for the original version of the questionnaire was 0.860 [14].
- Adherence to Refills and Medications Scale (ARMS), which is used to assess medication adherence [15]. ARMS consists of two domains: adherence to taking medications (8 items) and adherence to refilling prescriptions (4 items). It consists of 12 questions, with possible responses of “none”, “some”, “most” and “all the time”. The survey’s questions focused on the frequency of taking medications as prescribed, forgetting to take medications, deliberately not taking medications, forgetting to renew prescriptions, skipping medication doses without consulting a doctor, modifying medication doses on their own due to absentmindedness or well-being, postponing the purchase of medications due to their high price and buying medications to stock up. The questionnaire has a score range of 12–48, with higher scores indicating poorer adherence. The original ARMS correlated significantly with the Morisky adherence scale (Spearman’s rho = −0.651, *p* < 0.01). The ARMS correlated more strongly with measures of refill adherence than the Morisky scale [15]. Cronbach’s alpha for the Polish version of the questionnaire was 0.775–0.958 [16].

### 2.1. Statistical Methods

An analysis of quantitative variables (i.e., expressed in numbers) was carried out by calculating means, standard deviations, medians, quartiles, as well as minimum and maximum values. Qualitative variables (i.e., not expressed in numbers) were analysed by calculating the number and percentage of occurrences of each value. The values of qualitative variables were compared between groups using the chi-square test (with Yates’ correction for 2 × 2 tables) or the Fisher’s exact test when there were low expected values in the tables. The values of quantitative variables were compared between two groups using the Mann–Whitney U-test, whereas the Kruskal–Wallis test was used to compare the values of quantitative variables between three or more groups. When statistically significant differences were detected, a post-hoc analysis was performed using Dunn’s test in order to identify statistically significantly different groups. Correlations between quantitative variables were analysed using the Spearman’s correlation coefficient. A multi-factor analysis of the independent impact of a number of variables on a quantitative variable was performed using linear regression. The results are reported as the values of parameters of the regression model, with a 95% confidence interval. A level of significance of 0.05 was used in the analysis. Thus, all *p*-values of less than 0.05 were interpreted as indicating significant relationships. The analysis was performed using the R software, version 3.6.2 [17].

### 2.2. Ethical Considerations

The study was approved by the Bioethics Committee at the Wroclaw Medical University (approval no. KB-737/2018). All participants provided informed written consent after a thorough explanation of all the procedures involved. All patients were informed about the purpose and nature of the study and provided informed written consent to participate in the study. All patients completed all questionnaires. The study was conducted in accordance with the tenets of the Declaration of Helsinki.

## 3. Results

### 3.1. Sociodemographic and Clinical Characteristics of the Patients Studied

The socio-demographic characteristics of the patients studied are shown in Table 1. Most patients were in a relationship (73.54%), had secondary education (43.38%), lived in urban areas (63.08%) and were retired (48.31%). Forty per cent of patients were smokers. The largest proportion of respondents had been hospitalised once due to exacerbations of their disease (38.46%).

### 3.2. Medication Adherence (ARMS, TAI) and Beliefs about Medicines (BMQ)

The patients were uncertain about the overuse of medicines by doctors (a mean of 3.19 points per question) and about the harmful effects of medicines (a mean of 2.82 points per question) and did not know if they were concerned about taking their medication (a mean of 3.33 points per question) (Table 2A). The respondents tended to be convinced of the necessity of their medication (a mean of 3.87 points per question).

The patients reported moderate levels of overall adherence (21.15 ± 6.23), adherence to taking medications as prescribed (13.41 ± 4.44) and adherence to refills on schedule (7.74 ± 2.21). A total of 74.15% of patients demonstrated sporadic non-compliance, 59.69% exhibited deliberate non-compliance and 11.38% exhibited unconscious non-compliance with inhaled medications.

### 3.3. Impact of BMQ on ARMS and TAI

Belief that medicines are overused by doctors, belief that medicines are harmful and concerns about medicines were significantly (*p* ˂ 0.05) and positively (r ˃ 0) correlated with the total ARMS score and the two subscales of the ARMS. Therefore, the greater the belief that medicines are overused by doctors, the lower the level of adherence (the higher the score on the ARMS) in all dimensions (Table 2B). Belief that medicines are overused by doctors was significantly (*p* ˂ 0.05) and negatively (r ˂ 0) correlated with the lack of sporadic, deliberate and unconscious non-compliance. Belief in the necessity of medication was significantly (*p* ˂ 0.05) and positively (r ˃ 0) correlated with the lack of sporadic and unconscious non-compliance and was significantly and negatively (r < 0) correlated with the total ARMS score and the two subscales of the ARMS. Therefore, the stronger the belief in the necessity of medication, the higher the level of adherence (the lower the score) in all dimensions. Belief that medicines are harmful and concerns about medicines were significantly (*p* ˂ 0.05) and negatively (r ˂ 0) correlated with the lack of sporadic, deliberate and unconscious non-compliance. Therefore, the greater the concerns about medicines, the poorer the adherence to inhaled medications.

### 3.4. Regression Analysis—ARMS

The linear regression model showed that being unemployed (R = −5.073), being a non-smoker (R = −1.983) and belief in the necessity of medication (R = −0.34) are significant (*p* < 0.05) independent predictors reducing the total ARMS score (increasing the level of adherence) (Table 3A). In contrast, the number of hospital admissions due to exacerbations over the last year and belief that medicines are harmful (R = 1.897 and R = 0.417, respectively) increase the total ARMS score (reduce the level of adherence). The R^2^ coefficient for this model was 33.17%, which means that 33.17% of variation in the total ARMS score is explained by the variables included in the model. The remaining 66.83% depends on variables that were not included in the model, as well as random factors (Table 3A).

The linear regression model showed that being unemployed (R = −3.195) and belief in the necessity of medication (R = −0.34) are significant (*p* < 0.05) independent predictors reducing the ‘medication taking as prescribed’ subscale score, whereas the number of hospital admissions due to exacerbations over the last year (R = 1.364) and the belief that medicines are harmful (R = 0.278) significantly reduce the level of adherence to taking medications. The R^2^ coefficient for this model was 28.61%, which means that 28.61% of variation in the ‘medication taking as prescribed’ subscale score is explained by the variables included in the model. The remaining 71.39% depends on variables that were not included in the model, as well as random factors (Table 3A).

The regression analysis showed that being a working pensioner (R = −1.033), being an old-age pensioner (R = −0.711), being unemployed (R = −1.877), being a non-smoker (R = −0.825) and belief in the necessity of medication (R = −0.11) are significant (*p* ˂ 0.05) independent predictors in increasing the level of refill adherence. Belief that medicines are harmful is a predictor reducing the level of refill adherence (R = 0.14). The R^2^ coefficient for this model was 34.47%, which means that 34.47% of variation in the ‘refills on schedule’ subscale score is explained by the variables included in the model. The remaining 65.53% depends on variables that were not included in the model, as well as random factors (Table 3A).

### 3.5. Regression Analysis—TAI

The linear regression model showed that non-smoking status (R = 1.113) is a significant (*p* ˂ 0.05) independent predictor decreasing the level of sporadic non-compliance, whereas living in a rural area (R = −0.848) is a significant predictor increasing the level of sporadic non-compliance. The R^2^ coefficient for this model was 36.01%, which means that 36.01% of variation in the ‘sporadic non-compliance’ variable is explained by the variables included in the model. The remaining 63.99% depends on variables that were not included in the model, as well as random factors (Table 3B).

Independent predictors increasing the level of deliberate non-compliance are living in a rural area (R = −0.97), the number of hospital admissions due to exacerbations over the last year (R = −0.806), belief that medicines are overused by doctors (R = −0.211) and concerns about medicines (R = −0.115). Non-smoking status is a predictor decreasing the level of deliberate non-compliance (R = 1.956). The R^2^ coefficient for this model was 53.39%, which means that 53.39% of variation in the ‘deliberate non-compliance’ variable is explained by the variables included in the model. The remaining 46.61% depends on variables that were not included in the model, as well as random factors (Table 3B).

The linear regression model showed that belief in the necessity of medication is a significant (*p* < 0.05) independent predictor decreasing the level of unconscious non-compliance (R = 0.022). The R^2^ coefficient for this model was 21.67%, which means that 21.67% of variation in the ‘unconscious non-compliance’ variable is explained by the variables included in the model. The remaining 78.33% depends on variables that were not included in the model, as well as random factors (Table 3B).

## 4. Discussion

This study aimed at assessing adherence (using ARMS and TAI) to inhaled therapy of patients suffering from obstructive pulmonary diseases in relation to BMQ and sociodemographic data. We demonstrated that overall adherence to treatment was moderate and beliefs about medicines significantly impacted patients’ adherence. Being unemployed, being a non-smoker and belief in the necessity of medication were independent determinants of better medication adherence. On the contrary, the number of hospital admissions due to exacerbations of the disease over the last year and the belief that medicines are harmful were independent determinants of worse medication adherence.

Adherence rates among patients with asthma and COPD in daily clinical practice usually do not exceed 50% [18]. Data from observational studies reflecting real-life clinical practice conditions indicate that adherence rates in patients with obstructive pulmonary diseases usually range between 10% and 40% [19,20]. In the present study, patients reported moderate levels of adherence to inhaled therapy. Most of the patients exhibited sporadic non-compliance and as many as half of them exhibited deliberate non-compliance with inhaled medications. In a study by Duarte-de-Araújo on patients with COPD, 31.3% showed poor adherence to treatment, whereas 16.7% did not adhere to their inhaled therapy [12]. In the Thi study, the poor adherence in the COPD group and asthma group accounted for 63.9% and 50%, respectively [21]. In the Polish study by Polański et al., only 14.15% of patients with COPD demonstrated high adherence to medications [22]. Asthma patients tended to express a higher sense of satisfaction with inhaler devices than COPD patients [21]. The study by Plaza et el. also indicated that the asthma group was significantly more satisfied with inhaled therapy using an inhaler [23]. In the Sánchez-Nieto study, there were no significant differences in adherence between asthma and COPD patients at the start of the study, and the only predictor of low adherence was the gender of the participant being female [24].

Patients with polypharmacy have an increased risk of treatment side effects, which may lead them to believe that their treatment is not very effective or even harmful. Those patients who believe in the necessity of their medication for their health achieve much higher levels of adherence than those who do not hold that belief [9]. In the present study, patients who believed in the necessity of their medication reported better adherence compared to patients who did not believe their medication was a necessity. In a study by Duarte-de-Araújo, the mean BMQ—Necessity score was higher in those COPD patients who were adherent to their inhalation therapy [12]. In a study by Brandstetter et al., specific beliefs about the necessity of medication were positively associated with medication adherence both in patients with asthma and in patients with COPD, whereas general beliefs about the harmfulness and over-prescription of medicines had a negative impact on medication adherence only among asthma patients [11]. Thus, it can be assumed that in the case of most patients with asthma/COPD, beliefs that medicines are harmful or likely to cause side-effects may not be reflected in the patients’ actual medication-taking behaviour.

The findings from the present study showed that smokers are more likely not to adhere to treatment. Moreover, a regression analysis showed that being a non-smoker is an independent determinant of better adherence to medications. Similarly, a study by Duarte-de-Araújo found a statistically significant negative association between medication adherence and smoking. However, the study showed no significant association between demographic and clinical variables and adherence [12]. Patients with chronic conditions who are smokers are less likely than those who do not smoke to adhere to recommendations concerning the self-monitoring of their disease. In a study by Watson et al., smokers perceived more barriers and fewer benefits to adherence, compared with non-smokers [25]. This may suggest that smokers are not aware that it is smoking that has a negative impact on their health and believe that their health deteriorates due to the ineffectiveness of treatment.

Studies confirm that there is an association between increased adherence and the level of symptom control. There is an association between previous hospital admissions due to disease exacerbation and poorer adherence in patients with asthma and COPD. A retrospective study by Toy et al. showed that a five percentage point increase in the level of adherence resulted in a reduction in the number of hospital visits by 2.6%, reduction in the number of days spent in hospital by 3.1% and a reduction in the number of emergency room visits by 1.8% [26]. Increased adherence is associated with a reduction in hospital admission due to COPD, lower risk of severe exacerbations of COPD and a statistically significantly lower risk of death due to COPD [20]. High adherence (>80%) is associated with a significant reduction in the frequency of severe exacerbations in patients with COPD [20]. In the study by Nittala et al. asthma patients with high medication adherence had fewer emergency department visits (*p* = 0.0004) and hospital admissions (*p* = 0.0303) [27]. Observations from the 3-year TORCH (Towards a Revolution in COPD Health) clinical study showed that patients who do not adhere to medications have an over two times higher risk of death and an almost two times higher risk of re-admission to hospital [20]. In a study by Vestbo et al., the annual rates of hospital admission due to exacerbations were 0.15 and 0.27 for patients with good adherence and patients with poor adherence to medication, respectively [20]. Moreover, studies confirm that there is an association between better adherence and an increased level of asthma control and a reduction in the number of asthma-related hospital admissions and emergency department visits. The relationship between the level of adherence and the level of asthma control has been confirmed for different age groups (children and adults) and is similar to the association reported for COPD [28,29].

One of the factors identified in the literature as having an impact on adherence in patients with chronic conditions is economic inactivity. Our study found an association between being unemployed and better medication adherence in patients with obstructive pulmonary diseases. This finding is contrary to the findings reported in the literature. One of the reasons for non-adherence indicated by Haynes et al. [30] was financial status preventing patients from filling prescriptions. Patients with COPD often have to give up their work as their breathlessness prevents them from doing what they have to do for their job. In the study by Nittala et al., male and low-income patients with asthma had more emergency room visits [27]. In their study, Polański et al. found that economic inactivity had a significant adverse impact on adherence in patients with COPD [22]. Those patients who do not have a job (and thus have limited income) cannot afford to buy their medication, which has a negative impact on their adherence to treatment. On the other hand, as economically inactive patients have more free time and no obligations, they can devote more time to adhering to treatment recommendations relating to medication, doctor’s appointments, breathing exercises and check-ups. However, economically inactive patients have limited financial means to be able to adhere to complex treatment regimens. Poor adherence to treatment is associated with an increased number of exacerbations and makes it difficult to control disease symptoms, which indirectly affects the patient’s ability to work and study. Moreover, poor adherence is associated with increased work absence and increased frequency of short-term disability [31]. Further research is needed to gain a better understanding of the role these factors play in treatment adherence in COPD and asthma patients.

## 5. Study Limitations

Our study has several limitations. Medication adherence was assessed using self-rating tools. Thus, the assessment of the level of treatment adherence may not have been objective. ARMS does not appear to be validated against objective measurement of adherence (e.g., estimating proportion of days covered). In order to reduce bias resulting from social desirability, patients were informed that the study was anonymous. The lack of analyses in groups of patients differing in terms of their underlying disease may also be a limitation of the study. However, studies have shown that differences in treatment adherence and beliefs about treatment do not depend on sociodemographic and disease-related variables [9,12].

## 6. Practical Implications

Insufficient adherence to therapy in patients undergoing long-term treatment requires specific interventions. It is crucial to determine whether patients accept their treatment and to what extent they adhere to it. Ensuring that patients receive reasonable arguments supporting their treatment plan is essential to treatment success. This is particularly important in the case of older patients given their experience with and beliefs about medicines. Talking with patients about their concerns regarding medicines may also reduce the level of deliberate non-compliance. The evaluation of adherence to treatment and factors interfering with adherence should be used routinely in daily clinical practice.

## 7. Conclusions

1. Patients with obstructive pulmonary diseases are moderately adherent to inhaled medication.
2. Beliefs about medicines have a significant impact on adherence to inhaled medications.
3. Being unemployed, being a non-smoker and belief in the necessity of medication are independent determinants of better medication adherence to inhaled therapy. On the contrary, the number of hospital admissions due to exacerbations of the disease over the last year and belief that medicines are harmful are independent determinants of poorer medication adherence to inhaled therapy.

## Figures and Tables

**Table 1:** Sociodemographic and clinical characteristics of the patients studied.

Variable		Total (n = 325)
Age	Mean ± SD	63 ± 11
	Median	64
	Quartiles	57–70
Sex	Female	159 (48.92%)
	Male	166 (51.08%)
Marital status	Single	86 (26.46%)
	In a relationship	239 (73.54%)
Education	Tertiary	67 (20.62%)
	Secondary	141 (43.38%)
	Vocational	87 (26.77%)
	Primary	30 (9.23%)
Place of residence	Urban area	205 (63.08%)
	Rural area	120 (36.92%)
Professional status	Economically active	83 (25.54%)
	Working pensioner	25 (7.69%)
	Old-age pensioner	157 (48.31%)
	Disability pensioner	48 (14.77%)
	Student	1 (0.31%)
	Unemployed	11 (3.38%)
Smoking status	Regular smoker	83 (25.54%)
	Occasional smoker	47 (14.46%)
	Non-smoker	195 (60.00%)
Number of cigarettes smoked a day	1–4	54 (16.62%)
	5–9	46 (14.15%)
	10–14	18 (5.54%)
	Around 1 pack	11 (3.38%)
	More than 1 pack	1 (0.31%)
	Non-smoker	195 (60.00%)
Number of hospital admissions due to exacerbations over the last year	0	71 (21.85%)
	1	125 (38.46%)
	2–3	113 (34.77%)
	4–5	14 (4.31%)
	>5	2 (0.62%)
Comorbidities	Diabetes	149 (45.85%)
	Allergies	73 (22.46%)
	Heart and cardiovascular disease	139 (42.77%)
	Obesity	83 (25.54%)
	None	45 (13.85%)
Duration of disease	<1 year	9 (2.77%)
	1–4 years	68 (20.92%)
	5–10 years	122 (37.54%)
	>10 years	126 (38.77%)
Number of inhaled medications used	1	105 (32.31%)
	2	176 (54.15%)
	3 or more	44 (13.54%)
How many medication doses do you use a day?	One	32 (9.85%)
	Two	141 (43.38%)
	Three	68 (20.92%)
	More than three	81 (24.92%)
	I don’t know	3 (0.92%)
If applicable, why did you stop taking your prescribed medication?	Improvement	243 (74.77%)
	Lack of improvement	52 (16.00%)
	I felt unwell when taking it	59 (18.15%)
	Side effects	42 (12.92%)
	Fear of side effects	11 (3.38%)
	I did not buy the next dose	43 (13.23%)
	I did not have a prescription to continue the therapy	36 (11.08%)
	Lack of money	33 (10.15%)
	None of the above	70 (21.54%)
	I did not stop taking my medication	23 (7.08%)
Medications *	SAMA	84 (25.85%)
	SABA	43 (13.23%)
	LABA	206 (63.38%)
	LAMA	199 (61.23%)
	Theophylline	52 (16.30%)
	ICS	112 (34.46%)

SD—standard deviation; SAMA—short-acting muscarinic antagonist; SABA—short-acting β2-agonists; LAMA—long-acting muscarinic antagonist; LABA—long-acting β2-agonists; ICS—Inhaled glucocorticosteroids. * Percentages do not add up to 100, as this was a multiple-choice question.

**Table 2:** (**A**) BMQ, ARMS and TAI results; (**B**) Correlation analysis between beliefs about medicines (BMQ) and treatment adherence (ARMS, TAI).

**A—BMQ, ARMS and TAI results**						
**Questionnaire**				**Score Range**	**Mean per Question**	**Mean ± SD**
BMQ		Belief that medicines are overused by doctors		4–20	3.19	12.74 ± 3.18
		Belief that medicines are harmful		4–20	2.82	11.29 ± 2.70
		Belief in the necessity of medication		5–25	3.87	19.35 ± 2.97
		Concerns about medicines		5–25	3.33	16.67 ± 3.25
ARMS		Total ARMS score		12–48	1.76	21.15 ± 6.23
		Medication taking as prescribed		8–32	1.68	13.41 ± 4.44
		Refills on schedule		4–16	1.94	7.74 ± 2.21
						**%**
TAI *		Sporadic non-compliance				74.15%
		Deliberate non-compliance				59.69%
		Unconscious non-compliance				11.38%
**B—Correlation analysis between beliefs about medicines (BMQ) and treatment adherence (ARMS, TAI)**						
**Questionnaire**		**BMQ**				
		**Belief that medicines are overused by doctors**	**Belief that medicines are harmful**		**Belief in the necessity of medication**	**Concerns about medicines**
ARMS	Total ARMS score Medication taking	r = 0.301, *p* < 0.001 *	r = 0.382, *p* < 0.001 *		r = −0.167, *p* = 0.003 *	r = 0.317, *p* < 0.001 *
	Medication taking as prescribed	r = 0.281, *p* < 0.001 *	r = 0.361, *p* < 0.001 *		r = −0.179, *p* = 0.001 *	r = 0.304, *p* < 0.001 *
	Refills on schedule	r = 0.281, *p* < 0.001 *	r = 0.351, *p* < 0.001 *		r = −0.124, *p* = 0.026 *	r = 0.282, *p* < 0.001 *
TAI	Sporadic non-compliance	r = −0.351, *p* < 0.001 *	r = −0.366, *p* < 0.001 *		r = 0.132, *p* = 0.017 *	r = −0.334, *p* < 0.001 *
	Deliberate non-compliance	r = −0.441, *p* < 0.001 *	r = −0.435, *p* < 0.001 *		r = 0.072, *p* = 0.197	r = −0.48, *p* < 0.001 *
	Unconscious non-compliance	r = −0.21, *p* < 0.001 *	r = −0.245, *p* < 0.001 *		r = 0.153, *p* = 0.006 *	r = −0.186, *p* = 0.001 *

SD—standard deviation; TAI—Test of Adherence to Inhalers; *p*—correlation analysis; * statistically significant relationship (*p* < 0.05); ARMS—Adherence to Refills and Medications Scale; BMQ—Beliefs about Medicines Questionnaire; questions about the use of inhaled medications, the percentages do not sum up to 100%.

**Table 3:** Results of linear regression analysis (**A**,**B**).

**A**													
**Feature**		**ARMS**											
		**Total Score**				**Medication Taking as Prescribed**				**Refills on Schedule**			
		**Parameter**	**95%CI**		***p***	**Parameter**	**95%CI**		***p***	**Parameter**	**95%CI**		***p***
Sex	Female	ref.				ref.				ref.			
	Male	−0.588	−1.987	0.812	0.411	−0.3	−1.332	0.732	0.569	−0.288	−0.777	0.202	0.251
Age	[years]	−0.048	−0.12	0.024	0.191	−0.047	−0.1	0.006	0.082	−0.001	−0.026	0.024	0.945
Place of residence	Urban area	ref.				ref.				ref.			
	Rural area	1.248	−0.031	2.528	0.057	0.853	−0.09	1.797	0.077	0.395	−0.053	0.843	0.085
Marital status	Single	ref.				ref.				ref.			
	In a relationship	−0.122	−1.538	1.294	0.866	−0.344	−1.388	0.7	0.519	0.222	−0.274	0.717	0.381
Professional status	Economically active	ref.				ref.				ref.			
	Working pensioner	−1.512	−4.185	1.16	0.268	−0.48	−2.451	1.491	0.634	−1.033	−1.968	−0.097	0.031 *
	Old-age pensioner	−1.276	−3.293	0.742	0.216	−0.565	−2.053	0.923	0.457	−0.711	−1.417	−0.005	0.049 *
	Disability pensioner	0.356	−1.937	2.648	0.761	0.011	−1.68	1.701	0.99	0.345	−0.457	1.147	0.4
	Unemployed	−5.073	−8.607	−1.538	0.005 *	−3.195	−5.802	−0.589	0.017 *	−1.877	−3.114	−0.64	0.003 *
Smoking status	Regular smoker	ref.				ref.				ref.			
	Occasional smoker	−0.569	−2.596	1.459	0.583	−0.143	−1.638	1.352	0.851	−0.426	−1.135	0.284	0.241
	Non-smoker	−1.983	−3.688	−0.277	0.023 *	−1.158	−2.416	0.1	0.072	−0.825	−1.422	−0.228	0.007 *
Number of hospital admissions due to exacerbations over the last year	0	ref.				ref.				ref.			
	1	1.227	−0.444	2.897	0.151	0.925	−0.307	2.157	0.142	0.302	−0.283	0.887	0.312
	2–3	1.897	0.199	3.596	0.029 *	1.364	0.111	2.616	0.034 *	0.534	−0.061	1.129	0.079
	More than 3	1.865	−1.181	4.912	0.231	1.056	−1.19	3.302	0.358	0.809	−0.257	1.876	0.138
Duration of disease	Up to 5 years	ref.				ref.				ref.			
	5–10 years	0.683	−0.95	2.316	0.413	0.781	−0.423	1.985	0.205	−0.098	−0.669	0.474	0.738
	>10 years	1.299	−0.436	3.034	0.143	0.98	−0.3	2.259	0.135	0.32	−0.288	0.927	0.303
Do you know how to self-monitor your asthma?	Yes, definitely	ref.				ref.				ref.			
	Yes	0.214	−1.764	2.191	0.832	0.53	−0.929	1.988	0.477	−0.316	−1.008	0.376	0.372
	Uncertain	1.012	−1.276	3.3	0.387	0.716	−0.971	2.403	0.406	0.296	−0.505	1.097	0.469
	No/No, definitely not	1.4	−0.952	3.752	0.244	1.244	−0.49	2.979	0.161	0.156	−0.667	0.979	0.711
BMQ	Belief that medicines are overused by doctors	−0.105	−0.402	0.191	0.487	−0.085	−0.303	0.134	0.45	−0.021	−0.125	0.083	0.694
	Belief that medicines are harmful	0.417	0.053	0.781	0.025 *	0.278	0.009	0.546	0.043 *	0.14	0.012	0.267	0.033 *
	Belief in the necessity of medication	−0.34	−0.562	−0.117	0.003 *	−0.229	−0.393	−0.065	0.007 *	−0.11	−0.188	−0.033	0.006 *
	Concerns about medicines	0.175	−0.083	0.434	0.185	0.123	−0.067	0.314	0.206	0.052	−0.039	0.143	0.262
**B**													
**Feature**		**TAI**											
		**Sporadic Non-Compliance**				**Deliberate Non-Compliance**				**Unconscious Non-Compliance**			
		**Parameter**	**95%CI**		***p***	**Parameter**	**95%CI**		***p***	**Parameter**	**95%CI**		***p***
Sex	Female	ref.				ref.				ref.			
	Male	0.384	−0.247	1.014	0.234	0.226	−0.363	0.814	0.453	−0.075	−0.187	0.037	0.188
Age	[years]	0.023	−0.01	0.055	0.171	−0.007	−0.037	0.023	0.647	−0.001	−0.007	0.004	0.656
Place of residence	Urban area	ref.				ref.				ref.			
	Rural area	−0.848	−1.424	−0.271	0.004 *	−0.97	−1.508	−0.431	<0.001	0.049	−0.053	0.152	0.344
Marital status	Single	ref.				ref.				ref.			
	In a relationship	0.402	−0.236	1.04	0.218	0.358	−0.238	0.954	0.24	0.056	−0.057	0.169	0.334
Professional status	Economically active	ref.				ref.				ref.			
	Working pensioner	−0.964	−2.168	0.24	0.118	−0.221	−1.345	0.904	0.701	−0.036	−0.249	0.178	0.744
	Old-age pensioner	−0.291	−1.2	0.618	0.531	0.009	−0.84	0.858	0.984	0.077	−0.085	0.238	0.353
	Disability pensioner	−0.31	−1.343	0.722	0.556	−0.6	−1.564	0.365	0.224	0.035	−0.148	0.219	0.706
	Unemployed	0.347	−1.245	1.939	0.669	0.929	−0.558	2.416	0.222	0.168	−0.115	0.45	0.246
Smoking status	Regular smoker	ref.				ref.				ref.			
	Occasional smoker	−0.152	−1.066	0.761	0.744	0.402	−0.451	1.255	0.356	0.127	−0.035	0.289	0.125
	Non-smoker	1.113	0.344	1.881	0.005 *	1.956	1.238	2.673	<0.001	0.01	−0.126	0.147	0.881
Number of hospital admissions due to exacerbations over the last year	0	ref.				ref.				ref.			
	1	−0.036	−0.789	0.716	0.924	−0.806	−1.509	−0.103	0.025 *	−0.014	−0.148	0.119	0.832
	2–3	0.022	−0.744	0.787	0.956	−0.592	−1.307	0.123	0.106	0.02	−0.116	0.156	0.777
	More than 3	0.653	−0.719	2.025	0.352	−0.392	−1.674	0.89	0.549	−0.202	−0.445	0.042	0.106
Duration of disease	Up to 5 years	ref.				ref.				ref.			
	5–10 years	0.065	−0.67	0.801	0.862	−0.173	−0.86	0.514	0.623	0.014	−0.117	0.144	0.837
	>10 years	0	−0.782	0.781	0.999	0.167	−0.563	0.897	0.654	−0.023	−0.162	0.116	0.747
Do you know how to self-monitor your asthma?	Yes, definitely	ref.				ref.				ref.			
	Yes	−0.032	−0.923	0.859	0.944	0.001	−0.831	0.834	0.997	0.099	−0.059	0.257	0.222
	Uncertain	−0.258	−1.289	0.772	0.624	−0.478	−1.441	0.484	0.331	−0.023	−0.206	0.16	0.802
	No/No, definitely not	−0.513	−1.572	0.546	0.343	−0.665	−1.654	0.325	0.189	−0.005	−0.193	0.183	0.959
BMQ: Belief that medicines are overused by doctors		−0.122	−0.256	0.012	0.074	−0.211	−0.336	−0.086	0.001 *	−0.002	−0.026	0.022	0.872
BMQ: Belief that medicines are harmful		−0.112	−0.276	0.052	0.18	−0.044	−0.197	0.109	0.572	−0.017	−0.046	0.012	0.247
BMQ: Belief in the necessity of medication		0.067	−0.033	0.167	0.19	0.072	−0.022	0.165	0.134	0.022	0.004	0.04	0.017 *
BMQ: Concerns about medicines		−0.017	−0.133	0.1	0.78	−0.115	−0.224	−0.006	0.04	−0.002	−0.022	0.019	0.873

*p*—multi-factor linear regression; * statistically significant relationship (*p* < 0.05); ARMS—Adherence to Refills and Medications Scale; BMQ—Beliefs about Medicines Questionnaire.

## Data Availability

All the data are available from the corresponding author on request.
